# Prioritising quality traits for gender‐responsive breeding for boiled potato in Uganda

**DOI:** 10.1111/ijfs.14840

**Published:** 2020-10-30

**Authors:** Netsayi Noris Mudege, Sarah Mayanja, John Nyaga, Mariam Nakitto, Samuel Edgar Tinyiro, Damali Babirye Magala, Janet Cox Achora, Sarah Kisakye, David Bamwirire, Thiago Mendes, Tawanda Muzhingi

**Affiliations:** ^1^ International Potato Center Box 25171 Nairobi Kenya; ^2^ International Potato Center Ntinda II Road, Plot 47, Naguru Hill, Box 22274 Kampala Uganda; ^3^ Independent Consultant Old Naivasha Road Nairobi Kenya; ^4^ National Agricultural Research Laboratories P. O Box 7065 Kampala Uganda; ^5^ National Agricultural Research Organisation‐Mukono Zonal Agricultural Research and Development Institute P.O. Box 164 Mukono Uganda; ^6^ Ministry of Agriculture, Animal Industry and Fisheries (MAAIF) P.O Box 102, Plot 16‐18 Entebbe Uganda

**Keywords:** Breeding, gender, potato, quality traits, Uganda

## Abstract

Using quantitative, qualitative and sensorial data collected from western (Kabale) and central (Rakai) Uganda, this paper identifies and describes gender‐responsive traits preferred in varieties for the boiled potato market. These traits are aggregated into a product profile to support breeding programme design and decision‐making that will increase probability of variety acceptance. An interdisciplinary and participatory methodology was used to collect data on socio‐economic on trait preferences, processing and organoleptics and finally, to develop a lexicon through a sensorial panel. Characteristics that were important to both men and women, such as red skin and yellow flesh, are linked to market preferences. Women‐only preferred characteristics such as big size and mealiness are linked to processing efficiency and eating quality. Besides agronomic traits, breeders must consider factors such as gender roles, social norms, and market preferences traits that guide farmers and other food chain actors in their selection of new varieties.

## Graphical abstract

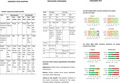

## Introduction

Potato is a key food security as well as a cash crop for smallholder farmers (Priegnitz *et al*., 2019, 2020) Thus, increased productivity may lead to improved farmers’ livelihoods and incomes (Mugisha *et al*., 2017). However, potato farmers can only achieve these benefits if the varieties they cultivate respond not only to their food needs but also to the needs of the market. The adoption rates for new potato varieties have been lower than expected. Potato breeding has focused on increasing yields and disease tolerance, while other traits preferred by men and women farmers and markets are given a low priority. Friedmann *et al.,* (2018), suggest that it is important to understand any gender differences in trait preferences because breeding programmes that do not take these differences into account may exacerbate gender inequalities and result in negative outcomes for women. Tufan et al., (2018) note that there is lack of information and tools to understand gender differences in trait preferences an understanding of these gender differences should influence breeding decisions1. This paper is based on work at the International Potato Center (CIP) and its partners on models and methods of gender‐responsive trait identification and gender‐responsive breeding. It contributes to the selection of traits for genomic selection and use of selection indices that aim to ensure that new varieties have wide and gender‐equitable impact.

The study on which this paper is based was implemented in Kabale and Rakai districts in Uganda, representative of major agroecological regions for potato production. Kabale district is located in south‐western Uganda. Potato is one of the most important crops grown. Rakai district is located in the central region of Uganda. Potatoes are the second most important food crop in the district after banana (matooke/cooking bananas) interms of production levels and economic value (Rakai District, 2014). The study selected boiled products because the most common mode of consuming potato in Uganda is in boiled form. In both Kabale and Rakai, potato is boiled together with beans, meat or groundnuts (*Katogo*), wrapped with cooking banana leaves and steamed or boiled and mashed before being consumed. The paper aims to identify and describe gender‐responsive boiled potato varietal traits to develop a product profile that addresses key traits required to replace key reference potato varieties in Uganda and to provide recommendations for breeding teams on gender‐responsive breeding. A product profile is a description of a variety with the necessary traits to replace existing or older varieties in a given market (Excellence in Breeding Platform).

## Materials and methods

### Study methods

The study which used both qualitative and quantitative methods was divided into three phases, to capture data across the food value chain in support of developing a food product profile for boiled potato. During the first phase, the research team collected socio‐economic and preference data in interviews with men and women farmers (seventy four men and sixty two women), traders (seventeen men and twenty women), vendors and restaurant owners (nine men and ten women), and 249 consumers (135 men and 114 women) to understand their trait preferences as well as why they prefer these traits. The team also interviewed four men and four women key informants. The research team conducted focus group discussions (FGDs) with sixteen men’s and sixteen women’s groups to collect information on community wealth ranking, livelihoods, preferred potato varieties and traits, as well as specific details about varieties and potato products. The team also collected information about varieties that men and women farmers cultivate, how farmers, traders and processors identify a good or bad potato for boiling, how the boiled potato is prepared and consumed; and what tradeoffs households make between the different uses of potato and its traits. Information from the respondents with regard to varietal preference during this phase was used in selection of varieties for processing diagnosis and consumer testing.

During the second phase, the study collected data from participatory processing and diagnosis of quality characteristics with four men and four women, as well as organoleptic data from consumers. Processors in each district were purposively selected to participate in the processor experiments. Each processor was given four potato varieties which they first assessed qualitatively and later proceeded to prepare and cook. In Rakai, processors evaluated the following varieties: Deo Deo (officially known as Rwangume or NAROPOT4) Kasumali, Victoria and Kabale. In Kabale district, processors evaluated the following varieties: Rwangume (NAROPOT4), Kinigi, Victoria and Kachpot 1. Using the descriptors generated in the first phase of the research, the research team refined and improved the protocols for organoleptic testing. A total of 114 women and 135 men from two rural areas (Rakai and Kabale) and two urban areas (Kyotera and Kabale) participated in organoleptic tasting. The same potato varieties used during the processing diagnosis in Rakai and Kabale were used for consumer tests. Potatoes were sourced from farmers and traders within the research area a day before conducting the consumer test activity.

The third phase was related to the *sensorial panel*. Since organoleptic testing is subjective and varies among individuals, we conducted descriptive sensory analysis with a trained panel comprising eleven members to identify objective well‐defined sensory descriptors suitable for boiled potato. The sensorial panel was implemented between the 11^th^ and 14^th^ of November 2019. This interpretation of consumer considerations into objective, measurable attributes facilitates understanding and communication with other actors by creating a liaison between consumers, scientists and breeders (Suwonsichon, 2019). All the descriptors used in the lexicon have corresponding technical definitions and are listed among the sensory vocabulary (ISO, 2008), except for the unique aroma and flavour characteristics. The panel was trained by both the International Potato Center and the National Agricultural Research Organization (NARO) staff in Uganda. Samples from six distinct genotypes, whose sensory characteristics varied enough to represent the entire product spectrum, were prepared and served to the sensorial panel for evaluation: Cruza, Kinigi, Victoria, NAROPOT 4 (Rwangume/Deodeo) and KACHPOT 1 and an unspecified watery variety. These varieties were obtained from farmers in the main potato growing regions of Uganda. Cruza was obtained from farmers in Mbale; Kinigi and KACHPOT 1 were obtained from farmers in Kabale, and NAROPOT 4 (Rwangume/Deodeo), Victoria and the watery variety were obtained from farmers in Rakai. All samples were harvested in October 2019, and the tubers were inspected to ensure no physical damage. The samples were then packaged in paper boxes and transported by road to the laboratories in Kawanda. During sample preparation, 2000 g of the sample potatoes were cooked until they could easily be penetrated with a toothpick or fork. Each panellist was presented with one tuber for evaluation. A lexicon (list of evaluated traits) was developed using quantitative descriptive analysis (Meilgaard *et al*., 2007; Swegarden *et al*., 2019). The descriptors in the lexicon were compared to the descriptors identified in the first phase of the study for similarities.

### Data analysis

FGD and KII data were coded and analysed. The principal investigator created a coding tree which was used to code data according to specific themes such as traits preferred by men and women farmers at different stages of the potato value chain, gender determinants of specific traits, social and economic determinants of trait preferences, gendered implications of preferred traits and the associated gender roles. Other codes to do with gender norms as well as division of labour and decision‐making were regarded as cross cutting themes in analyses. Data were analysed according to these themes. Survey data from Individual Interviews, traders and restaurants were analysed using Stata ver. 16. We extracted frequencies, percentages and proportions on how farmers, traders and processors identify a good or bad potato for boiling, how the boiled potato is prepared and consumed, what tradeoffs households make between the different uses of potato and its traits. These frequencies and percentages on various areas of interest were extracted from the quantitative data. Data from processors were analysed using Microsoft Excel.

Consumer data were analysed using XL STAT (2017 Addinsoft Inc, 244 Fifth Avenue, Suite E100 New York, NY, USA) using descriptive statistics for the JAR test. Hedonic data were analysed by ANOVA to compare overall liking of the test varieties; and multiple comparisons were used to separate the means. Differences were considered significant in cases where *P* < 0.05. We then summarised all this information to develop a gender‐responsive product profile.

## Results

### Main varieties used

The most common varieties cultivated by both men and women in Kabale were NAROPOT 4 (Rwangume), Kinigi and Victoria, while in Rakai, the most common varieties for both men and women were Deo Deo and Kabale. With the exception of Mbumba, which only women in Kabale cultivated, there was no significant difference between men and women with regards to varieties which they cultivated or regarded as important. The released variety NAROPOT 4, which went by the name Deo Deo in Rakai district and Rwangume in Kabale district, was the most popular variety in both study areas. However, other preferred varieties differed by region. Kinigi was cultivated only in Kabale district, while Kabale and Kasumali were only cultivated in Rakai, pointing to the need for enhanced understanding of geographical and regional differences that will help breeders develop and release materials that meet those market expectations.

The most important varieties were similar for both men and women in both regions. NAROPOT 4 was the most important variety for men and women in all regions. However, there were some variations in the second and third most important variety. For example, Mbumba was mentioned by women‐only, while only men mentioned NAKPOT 5 (Wanale) and Rutuku (Uganda 11).

In Kabale, we asked men and women in FGDs to share with us their preferred traits. Although women more than men mentioned cooking qualities, men and women had similar preferred traits. Both men and women were concerned about late blight, showing a preference for varieties that were tolerant. Some varieties such as KACHPOT 1 were not popular among farmers because of long dormancy. While overall, farmers were not happy with Victoria, they continued to cultivate it because of the short dormancy period, early maturity and big tubers, which was better for food security although the variety was not marketable and not tasty. A summary of the traits men and women mentioned for the different varieties is summarised in the Table 1.

**Table 1:** Cultivated varieties and their traits

Variety	Preferred traits by men	Preferred traits by women	Traits not preferred by women
Rwangume/NAROPOT 4/ (Kabale District)	It is tolerant to heavy rains Yields many tubers Uses little fungicide It is in high demand for chips	Mealy; Big tubers; It tolerates late blight; Many tubers; Quick maturing Marketable	Is now succumbing to late blight
NAROPOT 4 Deo deo (from Rakai District)	High yielding, mealy, disease‐resistant, marketable. Big size tubers, Good taste, Long shelf life (up to 3 months)	Mealy, Disease‐resistant, Marketable, Active buds – pink in colour, Mature, Smooth/shiny skin. Red skin, Yellow flesh	Rough skin: seeds do not grow when planted. Dead buds; Small size
Kinigi	Marketable	Has a high market, High income, uses little oil when making chips, is mealy	
Victoria	Big tubers, Grows well in the swampy areas, Early maturity, Short dormancy (sprouts fast)	Does not break after cooking, High yielding – has more than 7 tubers per plant, Early maturity (3 months after planting)	Susceptible to late blight, Requires lots of expensive fungicides. No market, Not tasty when cooked, Not mealy is watery. It is very hard when cooked, Withered leaves
KACHPOT 1	Mealy/high dry matter (good for chips) High yield, Big tubers, Disease‐resistant (especially to Late Blight)	Mealy	Soft skin (peels off easily) affecting marketability; A long dormancy, Only the wealthy households have this variety
Mbumba		Mealy, quick maturing, tolerates late blight, Cooks fast	
*Kasumali*	Early maturity (within 3 months), High yielding, Mealy	Early maturing Marketable and liked by people who make chips, Tasty, Firm in texture after cooking	Small tubers Rough, ugly skin Dead buds Needs new land every season, or wetland
Mabunda		Early maturing (2 months), High yielding, Big size tubers, Tasty, Mealy	Small tubers Shapeless
Kabale		Easy to get seed, Big size tubers, Food security	Very short shelf life, Not tasty, Watery

Men only listed least preferred traits for two varieties; hence, their results are summarised here instead of Table 1. In Rakai, the least preferred traits men mentioned for NAROPOT 4 (Deo Deo) were as follows: low shelf life when damaged in the field, disease prone when not sprayed, matured late (after 4 months) and hard both before and after boiling. Men did not like that Victoria was prone to disease, had bad taste and a glassy texture (*muwuutta*), rots easily (short shelf life, low storability) and watery when eating.

### Other socio‐economic determinants of trait and varietal preference

In FGDs, participants created a community wealth ranking ladder and then listed which varieties were preferred/cultivated by people at each stage, and why. Analysis of the data shows that there was a clear distinction between varieties preferred by the worse‐off and better off, except for Deo deo, which seemed to be versatile and acceptable by farmers across the social strata. Generally, more impoverished farmers preferred varieties and traits that do not need many inputs.

According to FGDs, Kasumali in Rakai, and Mbumba and Kimuli in Kabale were preferred by persons in the worse‐off step of the wealth ranking ladder as well as people in step 2 of the ladder (immediately after the impoverished step). ‘*We grow these varieties (Mbumba and Kimuli) because we do not have money to buy better varieties. These varieties are easy to manage and do not require crop protection measures and fertilisers. Even if we accessed the better varieties, we would still not be able to manage them'* (Women FGD, a participant who identified herself as belonging to the worse‐off group). While men do not cultivate worst performing varieties like Mbumba, women and poor households may cultivate them because they cannot afford the new varieties. Some varieties like Kimuli just germinate from tubers that are leftover in the field.

### Gendered quality characteristics of the raw crop (purchasing from the market)

Appearance traits seemed to be important for traders and consumers. Generally, big size tubers, potatoes that are firm when touched, are not damaged and have smooth skin were mentioned (see Table 2 for preferred qualities).

**Table 2:** qualities of a good potato for boiling when purchasing from the market

	Kabale %		Rakai %	
	Female (35)	Male (32)	Female (27)	Male (42)
Big size tubers	54.3	59.4	70.4	95.2
Smooth skin	5.7	21.9	59.3	45.2
No Damage	37.1	15.6	18.5	35.7
Firm in the hand	45.7	37.5	7.4	9.5
Not rotten	37.1	18.8	7.4	9.5
Red skin–Yellow flesh	20	21.9	7.4	11.9
Dust on the skin (to check if it was grown on good soil)	17.1	6.3	3.7	‐
No dents			7.4	11.9
White skin–yellow flesh	‐	3.1	7.4	7.1
Have Emerging sprouts which makes it sweeter	5.7	3.1	7.4	‐
Many eyes	‐	12.5		
Red skin–White flesh	2.9	3.1	‐	2.4
No sprouts	‐	3.1	‐	4.8
Fresh smell	‐	3.1	3.7	‐

When it came to marketed potatoes, 62.3% of women and 77.3% of men showed a preference for big size tubers. In the market, men and women stated that potatoes that are bad for boiling could be easily identified because they were damaged, had dead buds or eyes oozing a milky substance, and bad smell. This evidence suggests that breeders should be aware of the importance of market traits, post‐harvest handling traits that have an influence on freshness, shelf life and income potential for traders and consumers.

In FGDs, participants were asked for effects of top‐ranked traits on women's labour, decision‐making and control of income from the sale of potato ware. Big‐sized potatoes saved women labour in terms of peeling, while disease tolerance reduced women's labour demands related to spraying fungicides (see Table 3).

**Table 3:** Preferred traits and their implications for women

Trait	How to identify the trait	The benefit of the trait to women
Big‐sized tubers	The tuber is bigger than a fist or an egg When you put the tuber in your palm, you cannot close the palm 2 tubers should weigh a kilo 3 tubers can satisfy 5 people when cooked	Big‐sized tubers sell quickly in the market. They peel quickly and save labour A woman has control over what remains to be used for food. The big potatoes fill the basket quickly during harvest
Bright and shiny skin		This attracts buyers; they do not buy rough skin potatoes.
Nice buds and eyes		They attract customers directly increasing farmer income.
Red skin and yellow flesh	By looking and by pricking a small piece. When growing, the flower is pink. The buds are also pink	Its mealy It has a longer shelf life It is what buyers want.
Pest and disease resistance	The tuber has no signs of diseases, that is, no black spots, no visible rotting.	Disease resistance results in high yields thus fetching more income Labour for spraying is reduced. Disease‐resistant crops do not require a lot of spraying, therefore, saving on costs of agrochemicals and labour
High Yield	Has many eyes; can harvest many tubers from one plant Produce only big size potatoes A plant can have 15 tubers We can get approximately 5 bags from 1/8 of an acre.	
Early maturity	Matures in less than 4 months	Women can get money quickly to pay school fees; availability of food which stabilises the home.

Many of the preferred traits were related to market demand but had no direct observable impact on women. Women's ability to benefit from any of the marketable traits depends on household and community dynamics. For example, in relation to big‐sized potatoes or higher yields, some women in FGDs said, ‘*Some men will not agree with you on how to use the income. Yet you will have put in a lot of energy, and you feel demoralized*’. Men also agreed that women often did not control the income resulting from the higher yields. Decision‐making was not linked to traits but to the social‐cultural configurations of the society.

### Process description and diagnosis – processing quality traits

The raw material characteristics of potato tubers associated with good quality boiled potato by farmers included being firm when pressed, being hard when pressed by the hand and being heavy in weight. Farmers associated these characteristics of uncooked potato with mealiness after the potato is boiled. While some of the descriptors for farmers and processors were similar, processors also added good quality and shiny appearance, clear and smooth skin, not watery, not rotten and mature, no bad smell, tasty and not bitter as important indicators of a good potato for boiling (Table 4).

**Table 4:** Good and poor‐quality characteristics of raw potato by district, according to processors

Attribute category	Good quality		Poor quality	
	Kabale	Rakai	Kabale	Rakai
Skin colour	Red	Red		White, pale
Flesh colour		White, yellow	Grey ring	White
Tuber size	Big, medium, heavy	Heavy	Small size	Small size
Tuber texture	Hard/firm skin	Firm skin	Soft	Soft, watery
Skin surface	Shiny, not peeling off, smooth, not cracked, no holes	Shiny, smooth, no wrinkles, not cracked, good 'eyes' (not oozing), red 'eyes', shallow 'eyes.'		Rough, big deep eyes, oozing eyes, flaky skin.
Tuber shape		Long		
Condition of tuber	Not watery, not rotten, mature		Watery, rotten	
Taste			Bitter, not tasty	
Smell		No bad smell, fresh	Off‐flavour	No bad smell

While the processors in both districts mostly converged on the important characteristics to consider, processors in Rakai highlighted several characteristics concerning the ‘eyes’. Processors mentioned the following traits as not suitable for boiling: 'outer skin peeling off or flaky; tuber is not firm; tuber has cracks (*misheka*); potato not round in shape and small tuber size; and greyish ring inside the potato’.

Before cooking a good potato should not smell or should have what was regarded as a 'flat smell' and should have the smell of a fresh potato. Some respondents said that when cut, a good potato would smell like raw rice, or raw cabbage, sweet, fresh, have a natural smell, no smell or simply a nice smell (see Table 5).

**Table 5:** Descriptors of a good potato smell before cooking

Region	Kabale			Rakai		
Good Potato Smell	Female	Male	Total	Female	Male	Total
#	23	17	40	19	34	53
No smell	52.2%	64.7%	57.5%	10.5%	26.5%	20.8%
Good	13.0%	17.6%	15.0%	36.8%	17.6%	24.5%
Natural	4.3%	‐	2.5%	‐	2.9%	1.9%
Nice	21.7%	11.8%	17.5%	10.5%	2.9%	5.7%
Raw cabbage	8.7%	5.9%	7.5%	‐	‐	‐
Fresh	‐	‐	‐	63.2%	50.0%	54.7%
Raw rice	‐	‐	‐	‐	2.9%	1.9%
Sweet	‐	‐	‐	5.3%	23.5%	17.0%
Total	57.5%	42.5%	100.0%	35.8%	64.2%	100.0%

In contrast, respondents described a bad potato smell as, ‘like rotten vegetables’, 'a strong pungent smell', 'a smell similar to that of rotting cabbage', ‘a smell that remains on your hands even after washing them' or 'an offensive smell.' However, the traits of a bad potato smell are all associated with post‐harvest management practices as well as effects of diseases like late blight (rotten smell). Therefore, varieties that are resistant to late blight may also help prevent potatoes from rotting during post‐harvest storage. These are important observations that will help potato breeders in Uganda and east Africa.

Some studies have shown that flavours observed in cooked potato are related to several compounds inherent in the tubers or produced as a result of thermal treatments when cooking including hydrocarbons, terpenes, alcohols, an acid, furans, aldehydes, ketones, as well as halogenic, nitrogenous and sulphurous compounds (Bough, Holm & Jayanty, 2020). Moreover, the cooking method could modify the intensity of these flavours. When boiling, for example, some metabolites may leach out of the tubers thus reducing the intensity of the flavours. Additionally, these metabolites which indicate flavour have also been shown to correlate with texture, particularly mealiness. High throughput methods that quantify these flavour metabolites could thus be used to accelerate selection when breeding for flavour and texture improvement.

### Characteristics observed during processing boiled potato

During the processing diagnostics with the processors, we observed the variation of the technical aspects of cooking characteristics such as duration and yield at the various processing steps and how these varied among varieties. Processors in Kabale and Rakai followed the same procedure to prepare boiled potato. Potatoes were peeled and washed and then boiled.

Processors mostly preferred varieties whose peel was moderately soft with few eyes as the peeling yield was bound to be higher. For example, Kinigi, Kachpot1 and Rwangume were regarded to have moderately soft peel, which was also regarded as an indicator that the boiled potato would be tasty. KACHPOT 1 and Rwangume were also regarded as having a smooth and good peel appearance which was preferred. Processors regarded Victoria as watery in the hands and soft which was an indicator that it would not be mealy (a preferred characteristic) when boiled, so would not make a good boiled potato. Victoria was regarded as easy to peel; however, some processors in Rakai noted that Victoria has too many sunken eyes which made it difficult to peel and therefore not preferred for boiling. Farmers in Rakai regarded Kasumali as easy to peel owing to its moderately soft skin. During the peeling stage, Kasumali was assessed as good for boiling because it was firm when pressed with hands. It was also regarded as good because of its uniform yellow colour and no ‘eyes after peeling’.

Although Kabale was easy to peel, processors did not prefer its white flesh. They also regarded the peel as soft, and the potato looked watery (visible on the surface) which was interpreted to denote poor quality for boiling since it would be too soft after cooking. The results of Deo deo were contradictory. Some processors said it was not easy to peel, while others said it was easy. Some processors said it was not watery, so would be mealy when cooked while others said it had a lot of water and the colour was not the ‘good yellow colour’.

Processors mentioned that some varieties were easy to wash and were firm which was an indicator that they would make a good boiled potato. However, Victoria and Kinigi were regarded as soft and easy to wash, but processors feared that these would give a soft‐boiled potato which was not good. During washing, Rwangume and KACHPOT 1 were regarded as difficult to wash (remove all dirt) and were thus likely to give a good boiled product.

Good potatoes for boiling were said to take longer to cook because they are hard and mealy and produce a good aroma when cooked. In Rakai, Kasumali was regarded as good for boiling because it was not watery, looked mature because the skin was not peeling off although the peel is soft, easy to peel and smooth.

### Boiled/steamed potato descriptors

Except for appearance, where processors in Kabale preferred yellow colour, and processors in Rakai preferred yellow, white and cream colours of boiled potato, the good and poor‐quality characteristics of boiled potato were similar among processors in Kabale and Rakai. Processors associated good quality with cream, yellow or white‐fleshed potatoes that were soft‐firm, mealy, not watery, sticky and smooth in texture, with a good potato taste and aroma. Potatoes that were too soft, watery and not mealy in texture and those that lacked a good potato smell and taste were associated with poor quality. Potatoes that were bitter or tasteless were not regarded as having a good taste.

The most preferred variety in Kabale was Rwangume (NAROPOT 4) followed by Kinigi, KACHPOT 1 and Victoria, in descending order. Rwangume was liked for its colour (yellow), texture (sticky, smooth, firm, mealy) and good potato taste and smell while Victoria was disliked because it was deemed soft, bitter, white in colour and watery. In Rakai, Kasumali, whose characteristics were described as firm, mealy and yellow, was the most preferred. Victoria was the least preferred variety in Rakai for similar reasons as in Kabale.

In Kabale, the variety preference based on raw material and end product characteristics was different. While tubers of KACHPOT 1 were unanimously selected as the least preferred at raw material assessment, all processors agreed that Victoria had the least preferred end product.

### Ready‐to‐eat potato product quality characteristics

There were no major differences between men and women concerning preferred traits during consumption. In the survey, 72.6% of women and 62.2% of men preferred boiled potatoes that are mealy. A boiled potato was described as mealy if it looked powdery (*kukumuuka*), developed cracks on the surface after cooking, easily crumbled when rubbed between fingers, and felt dry and powdery the mouth when eating. About 62.9% of women and 52.7% of men mentioned aroma as a key preferred trait during consumption. Good aroma was described as potato odour (*akawowo*), a nice smell that induces appetite, aroma like boiled egg, a sweet garden smell and fresh smell. 38.7% of women and 24.3% of men mentioned taste as a key preferred trait during consumption. A good taste was described as ‘sweet’ taste (*kawooma*) and not bitter like the taste of potato that is left in the sun. 25.8% of women and 25.7% of men mentioned a potato being firm (hard when you touch by hand and in the mouth) as a key preferred trait when eating boiled potato. Softness was mentioned by 6.4% of women and 16.2% of men as a preferred trait. Softness was defined as *easy to chew and digest*. Participants reported that a soft potato can even be given to a patient recovering from illness to eat because of its ease to chew and swallow.

### Consumer preference for boiled potato

**Figure 1:**
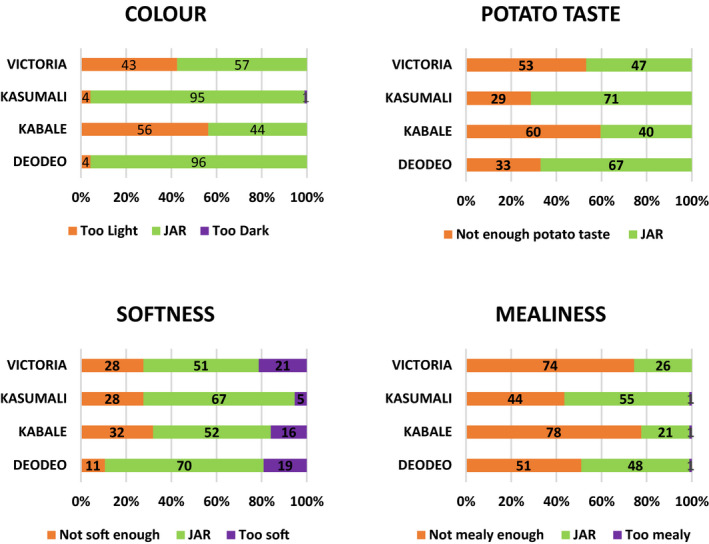
Consumer preference for potato varieties from Rakai: the JAR test.

**Figure 2:**
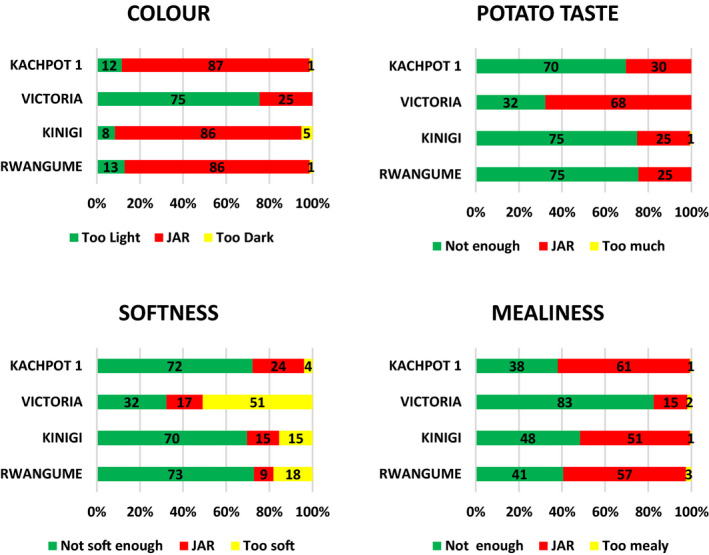
Consumer preference for potato varieties from Kabale the JAR test.

Results in Rakai from the overall liking on a nine‐point hedonic scale showed that Kasumali and Deodeo were the most preferred by consumers, followed by Victoria and Kabale. Overall liking of the most preferred varieties from Rakai was in the Hedonic region ‘Like slightly’. This could be attributed to their moderate mealiness as shown in the JAR test (Fig. 1). Similarly, the low overall liking of Victoria and Kabale could be attributed to their unsatisfactory level of mealiness with 74% and 78% of consumers, respectively, indicating they were not mealy enough. In Kabale, Rwangume and Kinigi were the most preferred varieties, and they were significantly different from Kachpot 1 and Victoria which was least preferred. Despite being ranked third overall, KACHPOT 1 was scored JAR by 87% (colour) and 61% (mealiness) of consumers had colour, taste and mealiness similar to or even better than Rwangume JAR test (86% colour and 57% mealiness) (Fig. 2).

*(Numbers inside bars show the percentage of respondents for each rating).

JAR test for varieties from Rakai is shown in Fig. 2. Deo Deo and *Kasumali* had the best colour, potato taste, softness and mealiness amongst the consumers. Kabale was least preferred for all the four attributes, and this was corroborated by the lowest overall likeability. With regards to varieties from Kabale, JAR test results revealed that colour preference and desired softness were highest for KACHPOT 1, Kinigi and Rwangume varieties (Fig. 2).

*(Numbers inside bars show the percentage of respondents for each rating).

Victoria had the most preferred potato taste, while KACHPOT 1 was best for mealiness. However, Victoria was the least liked for both colour and mealiness, which could explain why it was least liked overall.

### Gender‐responsive product profiles

Preference/non‐preference of good and poor characteristics was scrutinised for similarities and differences amongst men and women. As depicted in Table 6, there were characteristics that only women or only men mentioned for raw, during processing and boiled potato characteristics.

**Table 6:** Gender‐responsive product profile

Criteria	Good characteristics			Poor characteristics		
	Raw material	During processing	Boiled potato	Raw material	During processing	Boiled potato
Characteristics that were only mentioned by men	Red skin Yellow flesh	Not glassy	NR	Small size	Glassy (*muwutta*)Watery	Bad smell White colour Very soft
Characteristics that were only mentioned by women	No dents	Mealy	NR	Black spots	Black spots and holes Soft skin	Too flaky
Weighted characteristics by men	1. Big root size 2. Smooth skin 3. No damage	1. Firm skin 2. Yellow flesh colour 3. Not rotten	1. Good potato smell 2. Mealy 3. Good potato taste	1. Damaged root 2. Rotten 3. Dead buds or eyes	1. Small root size 2. Glassy (*muwutta*) 3. Watery	1. Watery 2. No potato taste 3. Not mealy
Weighted characteristics by women	1. Big root size 2. Smooth skin 3. No damage	1. Mealy 2. Big root size 3. Firm skin	1. Good potato smell 2. Mealy 3. Firm	1. Rotten 2. Damaged 3. Dead buds or eyes	1. Small root size 2. Has black spots 3. Soft skin	1. Not mealy 2. No potato taste 3. Watery

Big‐sized root and intact root with no damage were preferred raw potato characteristics for both men and women as well as for Rakai and Kabale districts. Firm skin (processing characteristic) good potato smell and mealy (final product characteristics) were also highly prioritised by both men and women. Less preferred raw and boiled potato characteristics were similar for both men and women although ranked differently. Importantly, overall, not much divergence was noticed between men and women for both preferred and less preferred characteristics.

### Guidance for breeders on developing consumer‐preferred varieties

The list of descriptors in the lexicon developed by the trained sensory panel, their definitions and scales is shown in Table 7. The panel identified 20 terms that described the sensory attributes of boiled potato and can help discriminate quality among genotypes. The terms include a number of aroma, appearance, flavour and texture attributes.

**Table 7:** Potato lexicon – Uganda

Descriptor	Technical definition	Panel definition (simplified definition)	Scale range and main anchors
Aroma			
Potato	Distinct aromatic notes associated with cooked potato	The distinct aroma of cooked potato	11 point scale, 0: odourless; 1: barely noticeable; 10: very strong
Green vegetable aroma	Distinct aromatic notes associated with cooked earthy vegetables like amaranth and beetroot	Distinct aroma of cooked amaranth and beetroot	11 point scale, 0: none; 10: very strong
Root tuber aroma	Distinct aromatic notes associated with cooked root crops such as sweet potato, cassava, and yam (*balugu*)	Distinct aroma of cooked root crops such as sweet potato, cassava, and yam (*balugu*)	2 point scale, 0: Absent; 1: Present
Cooked carrot	Distinct aromatic notes associated with cooked carrot	Distinct aroma of cooked carrot	2 point scale, 0: Absent; 1: Present
Appearance			
Yellow colour	Description of predominant colour of potato and its relative intensity	Intensity of yellow colour across the surface of the sample	11 point scale, 0: white; 5: cream; 10: yellow
Homogeneity of colour	Evenness of the distribution of the colour across the entire sample surface	Evenness of colour distribution across the sample surface	11 point scale, 0: highly variable; 10: consistent throughout
Translucency	Attribute of object that allows light to pass through it but not images to be distinguished	Quality of an object to allow light to pass through it but does not allow images to be distinguished such as a slice of steamed cucumber	2 point scale, 0: Absent; 1: Present
Flavour			
Potato	Flavour notes associated with cooked potato	Distinct flavour of cooked potato	11 point scale, 0: tasteless/bland; 1: barely noticeable; 10: very strong
Cooked carrot	Flavour notes associated with cooked carrot	Distinct flavour of cooked carrot	2 point scale, 0: Absent; 1: Present
Green vegetable	Flavour notes associated with cooked green vegetables such as amaranth and beetroot	Distinct flavour of cooked green vegetables such as amaranth and beetroot	2 point scale, 0: Absent; 1: Present
Root or tuber	Flavour notes associated with cooked roots or tubers such as sweet potato, cassava, and yam (balugu)	Distinct flavour of cooked root tubers such as sweet potato, cassava, and yam (*balugu*)	2 point scale, 0: Absent; 1: Present
Bitter aftertaste	Gustatory sensation that remains after swallowing product associated with the taste produced by dilute aqueous solutions of substances such as quinine	Lingering taste after swallowing that is similar to the taste of *nakati* (leaves of Ethiopian nightshade/mock tomato), *katunkuma* (bitter berries)	2 point scale, 0: Absent; 1: Present
Texture in mouth			
Fracturability	Mechanical textural attribute related to the force necessary to break into crumbs or pieces	Amount of mechanical textural force necessary to break the sample into distinct pieces	11 point scale, 0: deforms/does not fracture; 1: easily fractures; 10: needs high force to fracture
Hardness in mouth	Mechanical textural attribute related to the force necessary to achieve a certain deformation	Amount of force required to compress product between molars	11 point scale, 0: extremely soft, 10: hard
Crunchiness	Attribute of product to produce low pitched sound during rupturing	Production of low pitched sound while chewing certain foods such as cooked carrot, cooked cucumber	2 point scale, 0: Not crunchy; 1: Crunchy
Moisture (in the mass)	Textural attribute which describes the amount of water present in the sample mass	Amount of moisture present in sample mass	11 point scale, 0: dry; 10: extremely moist
Moisture release	Textural attribute related to release of moisture from a product when pressure is applied	Attribute of food products to release moisture when pressure is applied (when chewing) such as cooked cucumber	2 point scale, 0: Absent; 1: Present
Mealiness	An attribute associated with gumminess characterised by powdery mouthfeel	Perception of fine powdery particles upon chewing products such as egg yolk	11 point scale, 0: not mealy, 10: extremely mealy
Smoothness	Geometrical attribute associated with the overall degree of absence of particles within the sample	Degree of the absence of grainy particles in the mass sample	11 point scale, 0: grainy, 10: very smooth
Texture by hand			
Cohesiveness (mouldability)	Textural attribute relating to degree to which substance can be deformed before it breaks	Degree to which mass holds together when manipulated by fingers	11 point scale, 0: falls apart, 10: mouldable

The flavour and aroma attributes are, however, similar to those identified by Sharma (2019) for air fried and mashed potatoes made using potatoes in the United States of America. There are several reference foods common to Uganda's cuisine including balugu (yam), katunkuma (bitter berries), amaranth, nakati (leaves of Ethiopian nightshade/mock tomato), cassava and sweet potato that were cited by the panel. The attributes in the lexicon are comparable to the product profile for boiled potato. Mealiness (described as a powdery texture), hardness, bitterness, yellow colour, potato aroma and flavour are included in the lexicon having been mentioned by the consumers. Breeders can therefore use their definitions in the lexicon to understand the preferences of the consumers. In fact, some attributes such as hardness and yellow colour can easily be measured using laboratory instruments (Sato *et al*., 2017). Nonetheless, holistic characterisation of varieties based on the complete set of attributes in the lexicon is important since some varieties may have alternative uses beyond the scope of the established product profile for instance those that are highly cohesive (mouldable) may be useful for making mashed potatoes.

## Discussion

The discrepancy between variety preference at raw material and final product assessment, especially in Kabale, demonstrates the limitation of using raw material characteristics to anticipate the quality of cooked potatoes. To identify genetic traits to link to end‐user quality traits, it is more effective for breeding teams to assess traits of cooked products than those of raw potato. In cases where breeding teams choose to use characteristics of raw material they should validate these to ensure that they correctly predict characteristics of the product after cooking. This can be achieved by comparing biophysical and biochemical characteristics of the raw material to the sensory profile of the cooked product developed using established lexicon (Sato *et al*., 2017). Some traits of raw potato like hardness have already been linked to its texture after cooking (Ross *et al*., 2011).

Breeders should incorporate sensory quality characteristics in breeding selection protocols since varieties with superior yield and resistance characteristics may not always make superior products (Bechoff, *et al*., 2017). For example, although Kabale and Victoria varieties had higher processing yields in Rakai (probably owing to their size), they made the poorest quality boiled potato due to their undesirable sensory characteristics. However, breeding teams (which should be interdisciplinary) should use more objective means to standardise these sensory attributes. For example, the potato lexicon developed by the sensorial panel in Uganda complements the consumer surveys by providing clear definitions and reference foods for these terms and precisely translating hedonic or vaguely phrased traits such as ‘nice’ aroma, ‘bad’ smell, and ‘watery’. For example, moisture release and translucency in the lexicon are descriptors of a potato that is watery in nature. Expressions such as ‘sweet garden smell’ and ‘fresh’ smell from consumer surveys could refer to the earthy notes characteristic of the green vegetable aroma in the lexicon. In order to precisely match more of the vague consumer descriptors to those in the lexicon, food scientists in breeding teams should generate a sensory profile for the varieties studied in consumer surveys using the established lexicon. It is such information that breeders can use to develop medium to high throughput methods that can evaluate biochemical and biophysical measures associated with sensory quality to assist the breeding process.

Farmers and other chain actors have other considerations (such as gender roles and norms) besides agronomic traits that guide their selection of new varieties, which breeders must factor in product profile development. For example, in Uganda on one hand, the characteristics that were important to both men and women such as red skin colour and yellow flesh colour can be linked to preferred marketing attributes (also see Sanya *et al*., 2017; Elango & Kawarazuka, 2019). On the other hand, women‐only preferred characteristics such as big size and mealiness are linked to processing efficiency and eating quality and subsequently to their ascribed role of cooking and ensuring availability of good food in the home. Mudege *et al*. (2017) found that women prioritised culinary traits over disease resistance when selecting potato clones in a participatory variety selection exercise in Ethiopia. Elsewhere, Tufan *et al*., 2018 suggest that product design teams (which are multidisciplinary) should anticipate how the decisions they make will impact women’s labour, available resources and opportunities as these will determine whether new varieties are adopted or not.

A wide divergence between men and women was observed for quality processing traits: men ranked big‐sized tuber fifth while women ranked it second; and women ranked yellow flesh colour fifth, while men ranked it second. This implies that big size tubers would not be included in the priority characteristics for men, and neither would yellow flesh colour and red skin in the product profile for women. These are potential tradeoff issues which breeders may need to consider. Gender‐responsive breeding means providing farmers with a basket of choices so that traits that men and women value are included during product profile development (refer to Table 4 for traits to be included in the basket of choices). Similarly, mealiness was an important characteristic for women which was not mentioned by men, while not being ‘glassy’ was highlighted by only men. However, not being glassy and mealiness could be different ways of referring to mealiness which points to the need to harmonise the different vocabulary men and women may use to describe similar traits.

While for local potato varieties, there may have been regional variations in terms of preferred varieties, for improved varieties, Rakai and Kabale had similar preferences. Potato varieties with high consumer preference were balanced between local and improved; *Kasumali* (local) and Deo Deo (NAROPOT 4, improved) in Rakai, and Rwangume (NAROPOT 4, improved) and Kinigi (local) in Kabale. These are among the most commonly cultivated varieties in both locations, according to Namugga *et al.,* (2017). The most preferred varieties in Rakai also ranked best in terms of colour, taste, softness and mealiness (crumbly or powdery). Breeders could rank these quality traits highly in their breeding efforts. The variety Kabale was least preferred due to white colour, poor potato taste and lack of mealiness which were most critical attributes for consumer preference in Rakai. In Kabale, colour and mealiness were strong points for Rwangume and Kinigi; however, they were also deemed to be lacking in terms of characteristic potato taste and softness. Surprisingly, Victoria, which was least preferred overall, had the most satisfactory potato taste but was probably undone by white colour, being too soft and lacking mealiness. Therefore, consumer preference in Kabale was mainly driven by colour and mealiness as key attributes. Thus, our data suggest there is no need to develop different varietal profiles for the two regions; however, some specific preferences may need to be considered to ensure a basket of choices that meet the trait preferences of the target markets. Our findings help to shed more light regarding specific consumer‐preferred attributes.

## Conclusions

In conclusion, multidisciplinary breeding teams are an important part of potato product profile design. In addition to high productivity (on which breeders have traditionally focused), end users are also interested in the attributes such as agronomic management (especially how much external inputs are needed) as well as the sensorial quality of the end product in this case boiled potato. For instance, farmers in the low socio‐economic rank preferred varieties that do well with little or no external inputs. While there was not much difference between men and women in terms of sensorial attributes, gender differences were observed between men and women regarding processing attributes with women ranking attributes that are easier for processing highly than men. This was so because women are normally more engaged in processing than men. This shows the need for breeding teams to collect socio‐economic data to provide additional data points for breeding decisions. The need for such information entails that breeders cannot work alone but need to collaborate with social scientists, economists, nutritionists and other scientists. Product design teams should be multidisciplinary to gather all relevant information needed to understand trait preferences in order to develop relevant products. Potato breeding teams also need to rely more on traits of cooked products rather than raw potato qualities to make breeding decisions.

## Author Contribution

**Netsayi Noris Mudege:** Conceptualization (lead); Data curation (lead); Formal analysis (lead); Funding acquisition (lead); Investigation (lead); Methodology (equal); Project administration (lead); Resources (lead); Software (equal); Supervision (lead); Validation (lead); Visualization (lead); Writing‐original draft (lead); Writing‐review & editing (lead). **Sarah Mayanja:** Conceptualization (equal); Data curation (equal); Formal analysis (equal); Investigation (equal); Methodology (equal); Software (equal); Validation (equal); Writing‐review & editing (equal). **John Njuki Nyaga:** Formal analysis (equal); Writing‐original draft (equal); Writing‐review & editing (equal). **Mariam Nakitto:** Formal analysis (equal); Investigation (equal); Writing‐review & editing (supporting). **Samuel Edgar Tinyiro:** Formal analysis (equal); Investigation (equal); Writing‐review & editing (equal). **Damali Babirye Magala:** Investigation (equal); Writing‐review & editing (supporting). **Janet Cox Achora:** Formal analysis (supporting); Investigation (supporting). **Sarah Kisakye:** Formal analysis (supporting); Investigation (supporting). **David Bamwirire:** Investigation (supporting); Writing‐review & editing (supporting). **Thiago Mendes:** Funding acquisition (equal); Writing‐review & editing (supporting). **Tawanda Muzhingi:** Formal analysis (equal); Investigation (equal); Supervision (supporting); Writing‐review & editing (equal).

## Conflicts of Interest

The authors declare that they have no conflict of interest.

## Ethical clearance

We received ethical clearance from Makerere University School of Social Sciences Research and ethics committee.

## Data Availability

The data that support the findings of this study are available from the corresponding author upon reasonable request.
